# Aortic lumen repair with glue-felt technique before proximal anastomosis in acute type a aortic dissection surgery

**DOI:** 10.1186/s13019-024-03227-3

**Published:** 2025-01-08

**Authors:** Muhammed Varol, Berk Arapi, Çiğdem Tel Üstünışık, Deniz Göksedef, Suat Nail Ömeroğlu, Gökhan İpek, Ozan Onur Balkanay

**Affiliations:** 1 Department of Cardiovascular Surgery, Mayo Clinic, Rochester, MN USA; 2 Cerrahpasa Medical Faculty, Department of Cardiovascular Surgery, Istanbul University-Cerrahpasa, Istanbul, Turkey

**Keywords:** Aorta, Dissection, Cardiopulmonary bypass, Survival rate, Morbidity, Re-intervention, Propensity score matching

## Abstract

**Objectives:**

Despite the advances in medicine, aortic dissection remains a cardiac surgery emergency with high mortality and morbidity rates. This study examined the effects of the Glue + Felt technique, which uses biological glue and felt to repair the proximal anastomotic site, on the outcomes of patients with acute type A aortic dissection.

**Methods:**

A total of 108 patients who underwent surgery for acute type A aortic dissection at our clinic between 2007 and 2020 were included in the study. The patients were divided into two groups: the "Glue + Felt Technique" and the "Bentall-De Bono" groups, based on the surgical technique used for the aortic root. The effects of these two techniques on the development of intraoperative and postoperative complications and survival rates were statistically analyzed.

**Results:**

The Glue + Felt technique was used for 76 patients, while the Bentall-De Bono technique was used for 32 patients. The Kaplan–Meier analysis revealed significant differences in survival rates between the two groups over the entire follow-up period, both with and without propensity score matching (*p* < 0.001 and *p* = 0.02, respectively). However, no significant differences were observed in comparisons beyond the first 30 days of follow-up, either with or without propensity score matching (*p* = 0.573 and *p* = 0.561, respectively). The main factors contributing to this difference were the duration of cardiopulmonary bypass and aortic cross-clamp time (*p* < 0.05). During the average follow-up period of 46.2 ± 31.6 months, no re-intervention was required in patients from the Glue-Felt technique group.

**Conclusions:**

The mortality rate in aortic dissection surgery is higher with more extensive surgical intervention as the duration of cardiopulmonary bypass and aortic cross-clamp time increases. Repairing the lumen and reducing operation time in suitable patients using the Glue-Felt technique for the proximal anastomotic site positively impacts postoperative complications and improves in-hospital and 30-day survival rates, without increasing long-term re-intervention rates.

**Supplementary Information:**

The online version contains supplementary material available at 10.1186/s13019-024-03227-3.

## Introduction

Despite advancements in medicine and technology, acute aortic dissection remains a life-threatening emergency cardiac surgery, associated with high mortality and morbidity rates [1–3]. This condition involves a tear in the aorta's inner layer, leading to separation of its layers and necessitating prompt surgical intervention [4]. Surgical techniques vary depending on the dissection's location, extent of the intimal tear, and the involved part of the aorta and its branches [5]. Common methods in aortic dissection surgery include cardiopulmonary bypass (CPB), aortic cross clamp (ACC) time, hypothermia, and partial/total circulatory arrest [6]. However, deep hypothermia and prolonged circulatory arrest can lead to significant complications, including systemic inflammatory responses and platelet dysfunction [7–11]. Consequently, efforts have focused on minimizing hypothermia depth and shortening circulatory arrest and CPB durations [7, 9–13]. To achieve this goal, we used Teflon® (The Chemours Company, Wilmington, Del) felt and biological adhesive BioGlue® (CryoLife, Inc, Kennesaw, Ga) to repair the lumen in our clinic, which we believe shortens the duration of CPB and contributes to postoperative survival rates.

In this study, we examined the effects of the repair technique using biological glue and felt in the aortic root and proximal anastomotic site on outcomes in terms of survival and major complication rates of patients with acute type A aortic dissection (ATAAD).

## Patients and methods

Patients admitted to our tertiary care hospital between February 2007 and February 2020 with acute type A aortic dissection were retrospectively analyzed.

### Human ethics and consent to participate

Our study was conducted after the approval of the Local Clinical Research Ethics and Approval Committee (numbered 62,165,913–604.01.01–35805). Every human participant provided their informed consents in terms of involving the study. Our study was conducted in accordance with the Declaration of Helsinki.

### Pre-operative evaluation

Most patients were diagnosed with computerized tomography angiography, while others were diagnosed during catheterization or by transthoracic echocardiography. All diagnosed patients were urgently admitted to our Cardiovascular Surgery Intensive Care Unit. Hemodynamically stable patients with no signs of malperfusion underwent necessary blood preparations before surgery, while hemodynamically unstable or shocked patients were operated on urgently.

### Exclusion criteria

- Type-B aortic dissections
- Chronic aortic dissections
- Non-A Non-B aortic dissections
- Patients who required root replacement due to dissection but were eligible for valve-sparing surgery

### Clinical indications for the use of the glue-felt technique

In our study, the Glue-Felt technique was preferred for cases:

- Where the dissection did not extend to the level requiring aortic root replacement;
- Where the intimal tear did not progress into the sinuses of Valsalva;
- When the coronary ostia were intact;
- When the aortic valve was not severely affected to the point of causing significant insufficiency, but where the false lumen extended to the aortic root with intimal separation.

### Anesthesia monitoring

Patients were intubated with a single lumen endotracheal tube. Central venous pressure monitoring was performed on each patient, and invasive arterial monitoring from at least two arteries [both upper extremities (radial or brachial) or both upper and lower extremities (radial/brachial + femoral artery)] was done. Temperature was monitored with bladder and esophageal probes. In anuric patients with chronic renal failure, temperature was monitored additionally with a rectal probe. Standard inhalation anesthesia and narcotic agents were used in patients. Cerebral monitoring devices were routinely used during the entire procedure.

### Operative techniques

Median sternotomy was performed on the patients, and cardiopulmonary bypass (CPB) was initiated after systemic full heparinization. Alpha-stat pH management was used. The axillary artery was the most common site of cannulation, and alternatively, femoral artery cannulation was performed. Venous cannulation was performed from the right atrium, and a vent cannula was placed in the right superior pulmonary vein. Myocardial protection was achieved with both intermittent antegrade and continuous retrograde isothermic blood cardioplegia.

**Fig. 1:**
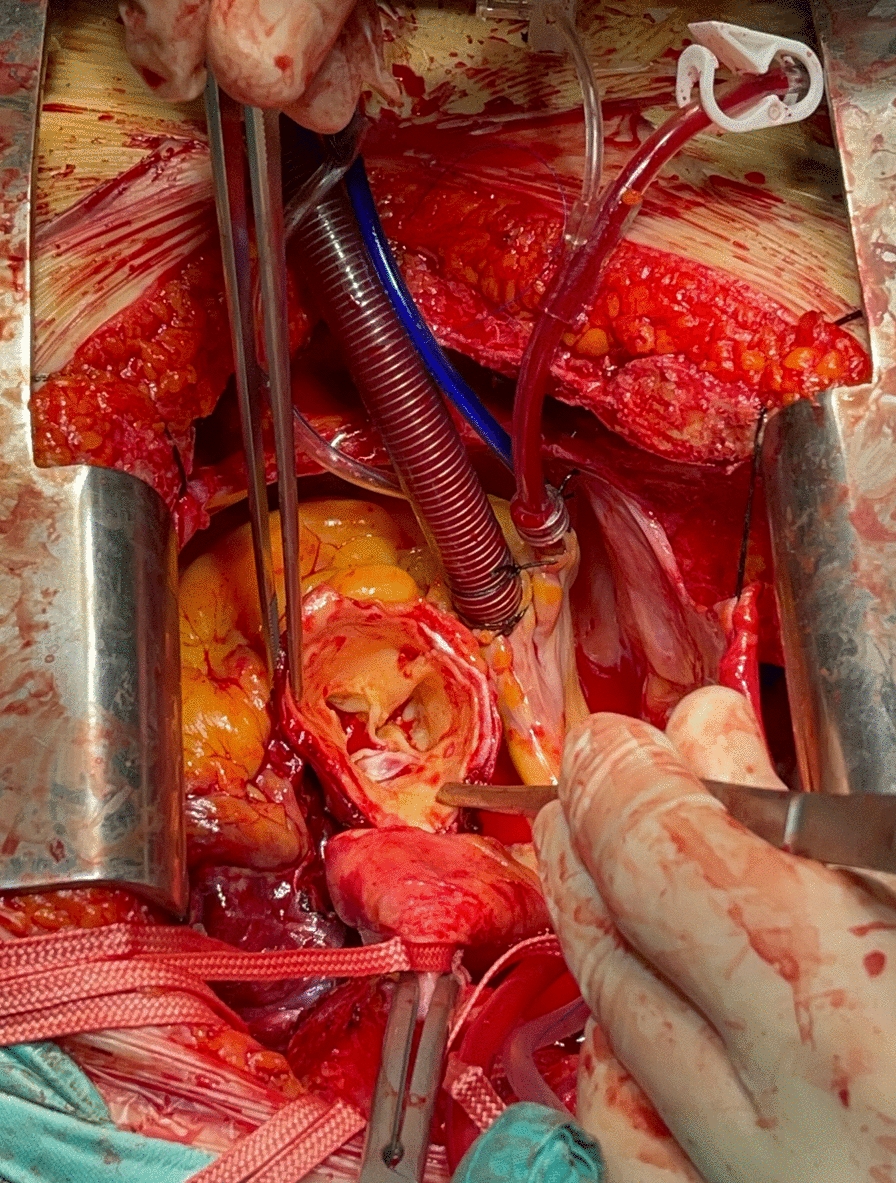
Glue-Felt technique utilized for the proximal anastomotic site

Patients underwent a cooling period of at least 50 min. During this time, repairs to the aortic root and ascending aorta were performed. After the aortotomy, selective antegrade cardioplegia was administered. The suitability of the ascending aorta and aortic root for repair was assessed, and Teflon® felt was inserted between the disintegrating lumens of the aortic root (if suitable for repair) to strengthen the aortic tissue and prepare it for anastomosis using the Glue-Felt technique for the proximal site (Fig. 1) (Movie 1). Additionally, topical cooling and pharmacological agents such as mannitol and propofol were applied.

During total arch replacement, antegrade cerebral perfusion was mostly used for brain protection, with retrograde cerebral perfusion rarely utilized. Antegrade cerebral perfusion was achieved via the right axillary artery, using an 8mm Dacron® graft end-to-side anastomosed to the artery, with occlusion of the left carotid and subclavian arteries to ensure a bloodless surgical field. Patients were cooled to a temperature of 18 °C.

Distal anastomosis was performed in all patients using an open technique. For patients who did not require arch reconstruction and were suitable for repair, Teflon® felt was placed between the disintegrating layers and reinforced with biological adhesives using the Glue-Felt technique for the distal site. For hemi-arch replacement, anastomosis was performed using 4/0 polypropylene sutures. During arch reconstructions, distal anastomosis was performed first under mostly antegrade cerebral perfusion, followed by anastomosis of the arch elements to the main graft using separate grafts. The same method was utilized during the elephant trunk (ET) procedure.

After completion of aortic arch reconstruction or hemi-arch replacement, arterial cannulation was performed from the branch of the main graft, and cardiopulmonary bypass was initiated, warming up the patient. Hyperthermia was avoided during this process, and the patient-perfusate temperature gradient was kept under 10 °C.

### Definitions

- Cardiogenic shock: A condition of low blood pressure that persists despite fluid replacement and is caused by heart failure.
- Transient neurological deficit: A temporary neurological disturbance, such as confusion, agitation, delirium, or motor deficit, that occurs after surgery.
- Persistent neurological deficit: A neurological disorder, either focal or global, that is confirmed by brain imaging and persists at discharge from the hospital.
- Operative mortality: The number of deaths occurring within 30 days after surgery.
- In-hospital mortality: The number of deaths occurring during hospitalization.
- Cardiovascular complications: Myocardial ischemia/infarction, low cardiac output, arrhythmia, and pericardial effusion/tamponade.
- Low cardiac output: A condition where the heart fails to pump enough blood to meet the body's needs, resulting in a cardiac output less than 2 L/min/m^2^.
- Respiratory complications: Pleural (pneumothorax and effusion) and parenchymal (atelectasis, pneumonia, edema, and acute respiratory distress syndrome) complications.
- Acute respiratory distress syndrome: A type of respiratory failure characterized by sudden onset, non-cardiogenic lung infiltration that can affect both lungs.
- Kidney Disease Outcomes Quality Initiative (KDOQI)—Acute Kidney Injury (AKI): Defined as an increase in serum creatinine (SCr) levels by 1.5 to 1.9 times the baseline, an increase of 0.3 mg/dL or more, or urine output less than 0.5 mL/kg/h for 6 to 12 h following surgery.
- Visceral organ complications: Mesenteric ischemia and mesenteric hemorrhage, acute hepatic injury, and hepatobiliary ischemia.
- The threat of rupture: It was identified by the presence of an advanced aneurysm associated with the dissection, hemorrhagic fluid due to pericardial/pleural leakage, and the presence of a significantly thinned dissection wall on tomographic examination.
- Post-operative severe bleeding: The need for a total blood transfusion of more than 10 units in the first 48 h after surgery.
- Requirement for blood product transfusion: Transfusion of more than 3 units of blood in the first 48 h after surgery.
- The indications for post-operative surgical revision due to bleeding were defined as: bleeding of 400 mL/hour for 1 h, 300 mL/hour for 2 h, 200 mL/hour for 4 h, or the presence of progressively increasing amounts of bleeding. Surgical revision was also performed in cases where vital and hemodynamic findings indicated cardiac tamponade, such as mediastinal widening on PA-chest X-rays or the presence of tamponade on echocardiographic examination.
- Systemic infection: Postoperative fever, leukocytosis, elevated C-reactive protein (CRP), and bacteremia indicating a systemic infection.

### Blood transfusion strategy

For post-operative patients, a Htc level < 26% or Hgb level < 8.5mg/dL was considered the anemia limit. Patients below this value received blood transfusions. During blood transfusions, the development of blood transfusion reactions was monitored by checking body temperature and continuous vital/hemodynamic parameters every 15 min.

### Extubation criteria

Patients were extubated if they met the criteria of the absence of any neurological deficit, stable hemodynamics, and a drainage amount less than 100 mL for two consecutive hours, except for those with an open sternum.

#### Statistical analysis

Demographic, pre-operative, intra-operative, and post-operative data of the patients were collected retrospectively and analyzed. Values were expressed as mean ± standard deviation or number and percentage (n, %). The Kolmogorov–Smirnov and Shapiro–Wilk tests were used to evaluate the distribution of continuous quantitative variables of the patients. Student's t-test was used to evaluate the quantitative variables with a normal distribution, and the Mann Whitney U and Wilcoxon Sum Rank tests were used for those without a normal distribution. The chi-square test was used for the analysis of qualitative variables, and Fisher's exact test was used in the presence of a low total frequency evaluation group. Kaplan Meier analysis was used for survival comparisons between groups. Propensity score matching was conducted between the groups to perform an additional survival analysis. Results with a p-value below 0.05 were considered statistically significant in comparisons.

## Results

A total of 108 patients were included in the study, with 76 patients treated with the Glue + Felt technique for the proximal site (Group-1) and 32 patients treated with Bentall-De Bono (Group-2). In terms of pre-operative demographic data, no significant differences were found between the groups, except for the presence of coronary artery disease, which was significantly higher in Group-2 (*p* = 0.049) (Table 1). According to EuroSCORE II calculations, the predicted operative mortality rates were similar between the groups (Group 1: 17.5% ± 11.1%; Group 2: 22.1% ± 10.8%; *p* = 0.054) (Table 1). Similarly, based on the German Registry of Acute Aortic Dissection Type A (GERAADA) scoring, the predicted 30-day/in-hospital mortality rates were comparable between the groups (Group 1: 16.6% ± 15.5%; Group 2: 20.4% ± 20.8%; *p* = 0.360) (Table 1).

**Table 1:** Demographic data of patients

Groups	Group-1^*a*^ (n = 76)		Group-2^*b*^ (n = 32)		
Parameters	n (%)	mean ± SD	n (%)	mean ± SD	p value
Age (years)		55.9 ± 12.6		56.6 ± 16.5	0.827
Gender (F)	21 (27.6)		12 (37.5)		0.309
NYHA		1.6 ± 0.9		1.6 ± 1.3	0.980
CAD	13 (17.1)		11 (34.4)		0.049^***c***^
PVD	5 (6.6)		2 (6.3)		1
CVD	1 (1.3)		2 (6.3)		0.209
Stroke history	8 (10.5)		2 (6.3)		0.720
COPD	7 (9.2)		2 (6.3)		1
LVEF (%)		55.3 ± 6.1		54.9 ± 8	0.784
Marfan/CTD	1 (1.3)		3 (9.4)		0.077
Cardiac surgery	12 (15.8)		3 (9.4)		0.545
EuroSCORE II (%)		17.5 ± 11.1		22.1 ± 10.8	0.054
GERAADA score (%)		16.6 ± 15.5		20.4 ± 20.8	0.36

*CAD: Coronary artery disease; COPD: Chronic obstructive pulmonary disease; CTD: Connective tissue disease; CVD: Carotid/Vertebral disease; GERAADA: German Registry of Acute Aortic Dissection Type A; LVEF: Left ventricular ejection fraction; NYHA: New York Heart Association functional classification; PVD: Peripheral vascular disease*

^***a***^*: "Glue-Felt technique for proximal site" group*

^***b***^*: "Bentall-De Bono" group*

^***c***^*: p* < *0.05*

Comparisons based on complaints and findings revealed that the threat of rupture was higher in Group-2 (*p* = 0.013) (Table 2). In Group 1, 55.3% of patients presented with chest pain, compared to 59.4% in Group 2 (*p* = 0.694), indicating no significant difference between the groups. Dyspnea was more common in Group 2, with 21.9% of patients experiencing this symptom compared to 6.6% in Group 1, which was statistically significant (*p* = 0.039). Syncope or stroke occurred in 5.3% of Group 1 and 18.8% of Group 2, with a trend toward significance (*p* = 0.062). Back pain was observed in 3.9% of Group 1 and 12.5% of Group 2, though the difference was not statistically significant (*p* = 0.192).

**Table 2:** Patient admission symptoms and observations

Groups	Group-1^*a*^ (n = 76)	Group-2^*b*^ (n = 32)	
Parameters	n (%)	n (%)	*p* value
Cardiogenic shock	5 (6.6)	6 (18.8)	0.080
Threat of rupture	12 (15.8)	12 (37.5)	0.013^***c***^
Penetrating ulcer/IMH	14 (18.4)	2 (6.3)	0.141
DeBakey Classification			0.144
Type 1	22 (28.9)	5 (15.6)	
Type 2	54 (71.1)	27 (84.4)	
Penn Classification			0.065
A	54 (71.1)	16 (50)	
B	17 (22.4)	10 (31.3)	
C	4 (5.3)	3 (9.4)	
B-C	1 (1.3)	3 (9.4)	

*IMH: Intramural hematoma; Penn: The University of Pennsylvania*

^***a***^*: "Glue-Felt technique for proximal site" group*

^***b***^*: "Bentall-De Bono" group*

^***c***^*: p* < *0.05*

There were no significant differences between the groups in terms of intra-operative perfusion values, including duration of brain and body circulatory arrest times, cerebral perfusion and hypothermia degrees, and total circulatory arrest times (*p* > 0.05) (Table 3). However, aortic cross-clamp time and cardiopulmonary bypass time durations were significantly higher in Group-2, which underwent root replacement, compared to Group-1 (105 ± 46, 149 ± 40 for aortic cross clamp time; 189 ± 55, 223 ± 65 for cardiopulmonary bypass time; respectively) (*p* < 0.05) (Table 3).

**Table 3:** Intraoperative hemodynamic parameters

Groups	Group-1^*a*^ (n = 76)	Group-2^*b*^ (n = 32)	*p* value
Parameters	mean ± SD	mean ± SD	
Cerebral-CA time (min)	17.8 ± 11.5	16.5 ± 6	0.441
Lower body-CA time (min)	42.8 ± 46.8	33.1 ± 41.5	0.327
Cerebral perfusion time (min)	33.7 ± 30.4	38.7 ± 20.3	0.702
Hypothermia (°C)	19.6 ± 2.7	20.4 ± 3.3	0.476
CPB (min)	189 ± 55	223 ± 65	0.006^***c***^
ACC (min)	105 ± 46	149 ± 40	< 0.001^***c***^

*ACC: Aortic cross clamp; CA: Circulatory arrest; CPB: Cardiopulmonary bypass*

^***a***^*: "Glue-Felt technique for proximal site" group*

^***b***^*: "Bentall-De Bono" group*

^***c***^*: p* < *0.05*

No significant difference was found between the groups in terms of distal anastomosis techniques (*p* > 0.05) (Table 4). However, there was a significant increase in coronary artery bypass grafting (CABG) in Group-2 compared to Group-1 (15 [46.9%] vs. 7 [9.2%], respectively) (*p* < 0.001) (Table 4). There was no statistically significant difference between the groups in the number of patients who underwent ET and frozen elephant trunk (FET) procedures (*p* > 0.05) (Table 4).

**Table 4:** Surgical procedures conducted

Groups	Group-1^*a*^ (n = 76)	Group-2^*b*^ (n = 32)	
Parameters	n (%)	n (%)	*p* value
CABG	7 (9.2)	15 (46.9)	< 0.001^***c***^
Distal anastomosis			
Hemi-arch	54 (71.1)	27 (84.4)	0.144
Total arch	22 (28.9)	5 (15.6)	0.144
*Islet*	5 (6.6)	1 (3.1)	0.667
*Multibranch*	17 (22.4)	4 (12.5)	0.237
ET	14 (18.4)	3 (9.4)	0.239
FET	2 (2.6)	3 (9.4)	0.153

*ET: Elephant trunk; FET: Frozen Elephant trunk; CABG: Coronary artery bypass grafting*

^***a***^*: "Glue-Felt technique for proximal site" group*

^***b***^*: "Bentall-De Bono" group*

^***c***^*: p* < *0.05*

In terms of post-operative morbidity data, there were no significant differences between the groups in terms of transient or persistent neurological deficits or cardiac morbidities such as low cardiac output syndrome, arrhythmia, and myocardial ischemia/infarction (*p* > 0.05) (Table 5). The rates of parenchymal complications in the lung, such as atelectasis, pneumonia, edema, acute respiratory distress syndrome, and pleural complications, such as pneumothorax and pleural effusion, were similar in both groups (*p* < 0.05) (Table 5). The rates of development of acute kidney injury were not statistically significant in both groups (*p* = 0.094), but the need for hemodialysis due to acute kidney injury was significantly higher in Group-2 (*p* = 0.027) (Table 5).

**Table 5:** Postoperative morbidity statistics

Groups	Group-1^*a*^ (n = 76)		Group-2^*b*^ (n = 32)		
Parameters	n (%)	mean ± SD	n (%)	mean ± SD	*p* value
Neurological deficit					
*Transient*	3 (3.9)		1 (3.1)		1
*Persistent*	4 (5.3)		2 (6.3)		1
Cardiac morbidities					
*MI*	0 (0)		1 (3.1)		0.296
*Low cardiac output syndrome*	23 (30.3)		15 (46.9)		0.099
*Arrhythmia*	25 (32.9)		15 (46.9)		0.170
Pericardial effusion/tamponade	16 (21.1)		12 (37.5)		0.075
Pulmonary complications					
*Parenchymal*^***c***^	15 (19.7)		12 (37.5)		0.107
*Pleural*^***d***^	5 (6.6)		2 (6.3)		1
Renal complications					
*Acute renal dysfunction*	25 (32.9)		16 (50)		0.094
*Hemodialysis*	10 (13.2)		10 (31.3)		0.027^***e***^
Mesenteric hemorrhage	1 (1.3)		0 (0)		1
Acute hepatic injury	1 (1.3)		1 (3.1)		0.507
Hepato-biliary ischemia	1 (1.3)		1 (3.1)		0.507
Post-operative severe bleeding^***f***^	15 (19.7)		16 (50)		0.002^***e***^
Wound complications	2 (2.6)		0 (0)		1
Systemic infection	20 (26.3)		12 (37.5)		0.245
RLN injury	3 (3.9)		0 (0)		0.553
Blood transfusion^***g***^	37 (48.7)		24 (75)		0.012^***e***^
IABP/ECMO	5 (6.6)		8 (25)		0.019^***e***^
ICU (days)		6.5 ± 10.3		9 ± 10.2	0.240
Length of stay (days)		15.5 ± 16.7		15.9 ± 15.8	0.914
Re-admission to hospital	5 (6.6)		5 (15.6)		0.158
Re-intervention^***h***^	0 (0)		2 (6.3)		0.086

*IABP/ECMO: Intraaortic balloon pump/ extracorporeal membrane oxygenation; ICU: Intensive care unit length of stay; LCOS: Low cardiac output syndrome; MI: Myocardial ischemia/infarction; RLN: Recurrent laryngeal nerve*

^***a***^*: "Glue-Felt technique for proximal site" group*

^***b***^*: "Bentall-De Bono" group*

^***c***^*: Atelectasis, pneumonia, edema, acute respiratory distress syndrome*

^***d***^*: Pneumothorax, pleural effusion*

^***e***^*: p* < *0.05*

^***f***^*: The indications for post-operative surgical revision due to bleeding were defined as: bleeding of 400 mL/hour for 1 h, 300 mL/hour for 2 h, 200 mL/hour for 4 h, or the presence of progressively increasing amounts of bleeding*

^***g***^*: Use of* > *3 units of blood product*

^***h***^*: Due to aortic root abscess/pseudoabscess or aortic valve surgery*

Regarding post-operative bleeding rates, it was higher in Group-2 (*p* = 0.002), and the need for blood transfusion was significantly higher in this group (*p* = 0.012) (Table 5). The need for circulatory support systems was also higher in Group-2 compared to Group-1 (*p* = 0.019) (Table 5). There was no difference between the two groups in terms of follow-up periods in the intensive care unit, hospitalization times, and re-admission after discharge (*p* > 0.05) (Table 5). Mesenteric ischemia and spinal cord damage were not detected in either group.

**Fig. 2:**
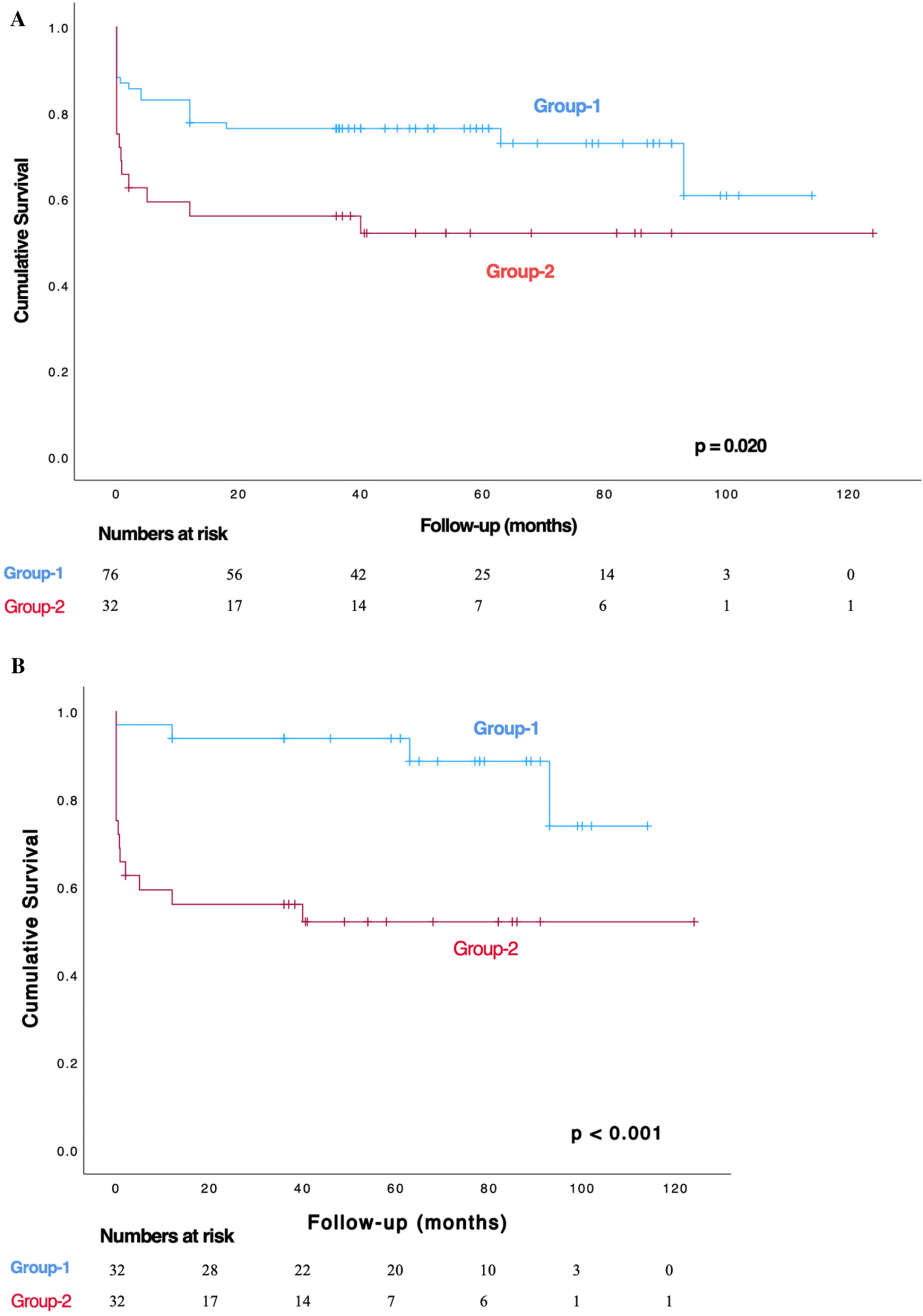
**A** Kaplan–Meier survival curves comparing the groups over the entire follow-up period.** B** Kaplan–Meier survival curves comparing the groups over the entire follow-up period after propensity score matching

**Fig. 3:**
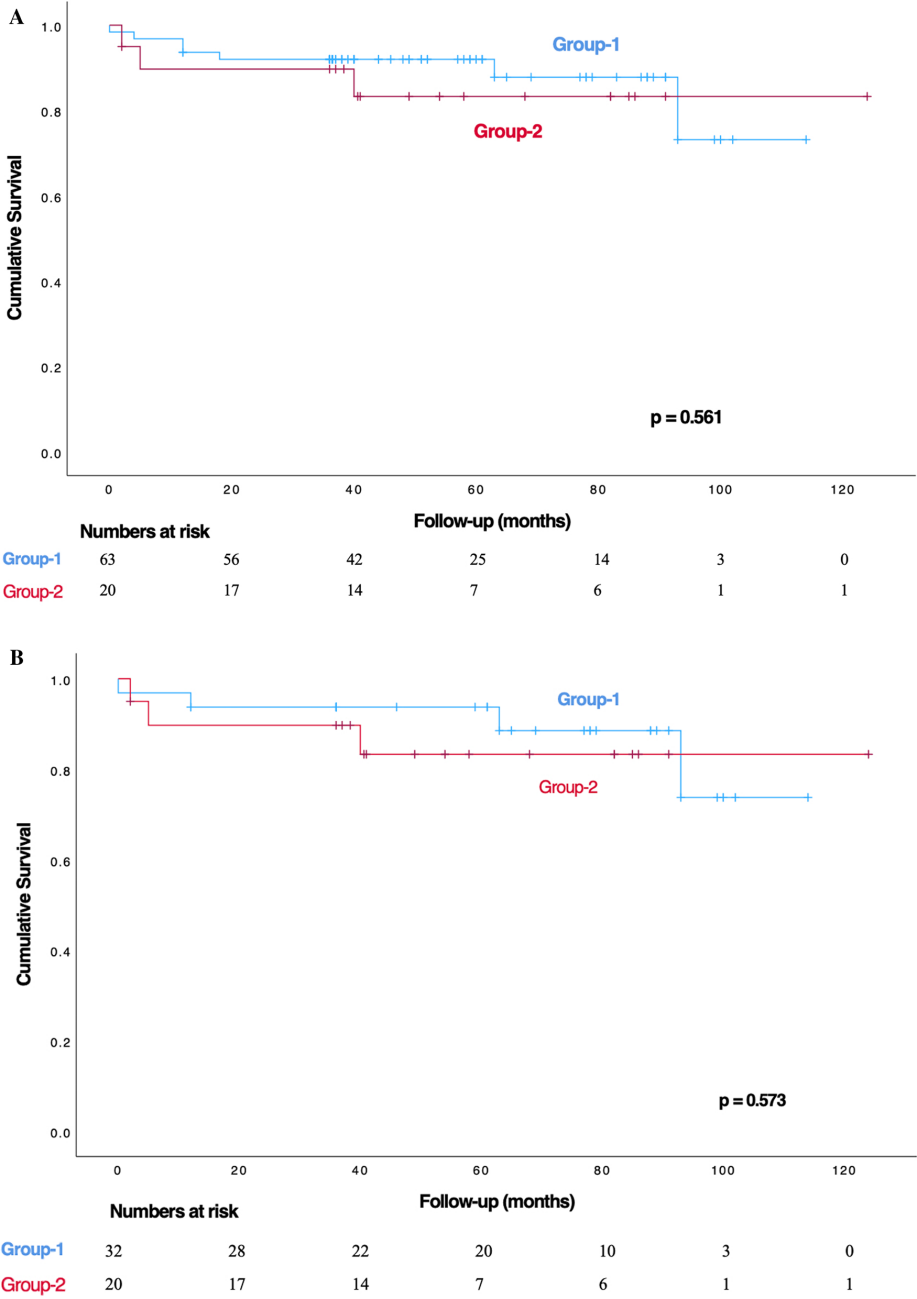
**A** Kaplan–Meier survival curves comparing the groups over the follow-up period beyond the first 30 days.** B** Kaplan–Meier survival curves comparing the groups over the follow-up period beyond the first 30 days, after propensity score matching

In the cumulative survival evaluation, there was a significant difference in survival during the early post-operative period (within the hospital and in the first 30 days) (*p* = 0.034). However, no significant change was observed in mortality rates after discharge (*p* > 0.05) (Table 6). The mean follow-up times for Group-1 and Group-2 were 46.2 ± 31.6 and 34 ± 36 months, respectively. The one, three, and five-year survival rates for Group-1 were 82.9 ± 4.3%, 76.3 ± 4.9%, and 76.3 ± 4.9%, respectively. For Group-2, the one, three, and five-year survival rates were 59.2 ± 8.7%, 55.9 ± 8.8%, and 51.9 ± 9.1%, respectively. The Kaplan–Meier analysis revealed significant differences in survival rates between the two groups over the entire follow-up period, both with and without propensity score matching (*p* < 0.001 and *p* = 0.02, respectively) (Figs. 2A, B). However, no significant differences were observed in comparisons beyond the first 30 days of follow-up, either with or without propensity score matching (*p* = 0.573 and *p* = 0.561, respectively) (Fig.3A, B).

**Table 6:** All-cause mortality rates across groups

Groups	Group-1^*a*^ (n = 76)	Group-2^*b*^ (n = 32)	
Parameters	n (%)	n (%)	*p* value
In-hospital^***d***^	14 (18.4)	12 (37.5)	0.034^***c***^
After discharge	6 (7.9)	3 (9.4)	1
First 30 days^***d***^	14 (18.4)	12 (37.5)	0.034^***c***^

^***a***^*: "Glue-Felt technique for proximal site" group*

^***b***^*: "Bentall-De Bono" group*

^***c***^*: Cumulative mortality values*

^***d***^*: p* < *0.05*

In the follow-up of the cases in Group 1, where the Glue-Felt technique was applied, none of the five patients (6.6%) who returned to the hospital required any interventions related to the aortic root or native aortic valve, such as aortic root pseudoaneurysm or additional dissection of the aortic root. It was observed that during the average follow-up period of 46.2 ± 31.6 months, there was no need for re-intervention in these patients.

## Discussion

Aortic dissection remains a critical emergency in cardiovascular surgery, with high morbidity and mortality rates despite advancements in medical care [2–4, 14]. Emergency surgery is required for patients diagnosed with ATAAD if there are no signs of severe neurological damage or malperfusion [4]. Regardless of the surgical technique used, the primary treatment goal should be to ensure the patient's survival [15]. In ATAAD treatment, minimizing deep hypothermia and shortening CPB and ACC times are crucial for reducing morbidity and mortality [2, 3]. The surgical technique to be used varies based on the regions affected by the dissection and the exclusion of the entry tear and malperfusion play important roles [16]. Various techniques and their combinations have been previously defined [17–25]. The Glue-Felt technique, which involves using biological adhesive and felt strips to repair the aortic root, has been introduced as a method to decrease CPB and ACC durations [25]. During the operation, we attempted to use techniques that would shorten the duration of deep hypothermic circulatory arrest and cardiopulmonary bypass [26]. Both the proximal aorta and distal arch extensions of the dissection were assessed for this purpose. The Glue + Felt technique, a lumen repair technique, was used at appropriate anastomotic sites. For unsuitable patients, the Bentall-De Bono technique was used for the proximal part, and total arch replacement and elephant trunk/FET techniques were preferred for the distal part. In our study, there were no significant differences in terms of different interventions made to the distal aorta in both groups (*p* > 0.05) (Table 4). When intra-operative perfusion data were evaluated, no significant differences were found in the duration and degree of hypothermia. Furthermore, one of the key postoperative morbidity and mortality determinants, and a primary target for reduction in new surgical techniques, is total circulatory arrest time [3]. It is worth noting that no significant differences were found between the two techniques concerning this parameter, particularly in relation to distal anastomotic involvement. Although there were no distinct differences in terms of distal intervention types and related parameters such as total circulatory arrest and hypothermia, the statistically significant differences in CPB and ACC times between the groups could become more advantageous in more complex interventions, such as total arch replacement or distal ET applications. These more extensive interventions, where distal anastomotic areas require additional attention, might further highlight the advantages of the Glue-Felt technique in managing these complex cases. From a fundamental surgical perspective, our approach of not attempting to replace the entire dissected aorta but instead focusing on ensuring stabilization with minimal intervention while repairing or replacing the affected regions reflects a core principle. Balancing both proximal and distal anastomoses in such situations can sometimes yield suboptimal results in clinical practice. As John Elefteriades famously said, "The goal of surgery is to leave the operating room with a live patient." In this context, regardless of surgical complexity or challenges, the ultimate objective is to ensure the patient’s survival. We believe that the greatest advantage of the Glue-Felt technique, when indicated, is its ability to facilitate proximal repair, allowing us to allocate more time and effort to addressing distal issues. Therefore, we believe that the reduction in CPB and ACC times provides a distinct advantage in such cases.

As aortic dissection is a dynamic event that can occur in any part of the aorta, clinical symptoms will vary depending on the location and extent of the dissection [27]. In patients who visited our clinic, back pain, particularly chest pain, was the most common symptom (Table 2). In parallel with the presenting symptoms, CABG rates were higher in Group-2 (*p* < 0.001) (Table 4). This was likely due to the high number of patients with coronary artery disease in Group-2 and the possibility of coronary dehiscence occurring in root destructions that cannot be repaired. In our study, since the cases involved acute aortic dissection and required emergency intervention, routine preoperative coronary angiographic imaging was not obtained. Therefore, the diagnosis of coronary artery disease was based on preoperative history, findings from prior coronary angiography, and intraoperative manual palpation of the coronary artery course, with the detection of significant plaque. CABG was performed in cases where there was a history of known coronary artery lesions or when intimal tears in the coronary ostium were present at a level that could not be addressed with replacement. Since involvement of the coronary ostium falls outside the indications for using the Glue-Felt technique, it is naturally expected that CABG was performed more frequently in Group 2 patients, who underwent the modified Bentall-De Bono procedure. As previously reported in the literature, CABG is one of the leading factors that effect the mortality rates after ATAAD surgery [3]. To minimize the impact of pre-existing CAD differences on postoperative mortality rates, we performed propensity score matching to further compare survival rates between the groups (Figs. 2B and 3B). These comparisons also demonstrated a significant reduction in 30-day mortality rates (Fig. 2B).

The management of hypothermia during aortic dissection surgery is crucial, as deep hypothermia can lead to complications such as systemic inflammatory responses and platelet dysfunction [3, 7, 9–11]. Recent researches have explored the use of moderate hypothermia circulatory arrest (MHCA), assessing its safety and efficacy [7, 9, 11]. A study by Wakisaka et al. evaluated MHCA in 358 patients undergoing hemiarch replacement for ATAAD and found it to be a viable strategy with acceptable outcomes [11]. Additionally, the duration of hypothermic circulatory arrest (HCA) has been identified as a significant factor affecting prognosis in aortic arch surgery [3, 10]. Song et al. emphasized the importance of controlling HCA duration during aortic dissection surgery, suggesting that prolonged HCA times are associated with adverse outcomes [10]. To achieve this goal, a modified Glue-Felt technique also showed promising results at the distal anastomotic site [24]. A study by Takago et al. described a novel reinforcement method using pre-glued felt strips with Hydrofit® for distal anastomotic sites in acute type A aortic dissection, achieving successful outcomes without intraoperative bleeding at the anastomosis site [24]. Since our technique primarily involves an intervention related to the aortic root, it has offered a statistically significant reduction in CPB and ACC times. However, no direct reduction effect was observed on both cerebral and lower body arrest times. The fact that there was no difference in deep hypothermia levels and circulatory arrest times between the two groups, which are more closely related to the distal aortic pathology and anastomosis types, and the extent of distal involvement of the aortic dissection, indicates that the focus can be shifted to the differences in the proximal stage, disregarding the distal aortic bed interventions, which do not create a difference in survival addressed in the main study. In the surgical treatment of aortic dissections, one of the primary goals is to minimize the level and duration of deep hypothermia, which is one of the most significant factors impacting the higher morbidity and mortality rates compared to other cardiac surgical interventions. In the cases included in this study, the method preferred during the period in which we performed aortic surgery was aimed at reducing the ischemic effects on the body's organs, particularly the kidneys and intestines, in situations where it was not clear whether the continuation of the intimal tear involved the aortic arch and its branches before the removal of the aortic cross-clamp. In cases where the necessity for separate anastomoses of the arch elements could arise, and where the total body arrest time could be prolonged, the patient was cooled to minimize these risks. Even when the surgical setup allowed for antegrade cerebral perfusion and axillary cannulation, and the aortic arch elements were controlled, hypothermia was preferred to reduce ischemic effects on the body in cases of total arch replacement, where the ischemic duration of the lower body could be longer. In the aortic dissection surgeries we perform today, however, we aim to minimize this time further and reduce the need for hypothermia [2–4]. In cases where there is a high likelihood of aortic arch dissection and involvement of the arch elements, and where the arch elements are pre-anastomosed, thereby reducing the duration of distal anastomosis despite total arch replacement, we no longer prefer deep hypothermia levels [7, 9–11]. In cases where dissection of the distal thoracic aorta and abdominal aorta is present, we previously preferred hypothermic status to reduce the level of ischemic effects on the body, as we avoided femoral cannulation due to the risk of provoking retrograde flow and dissection. What was essentially meant is that in cases where total arch aortic replacement is required due to distal involvement, the Glue-Felt technique, considered a faster form of the procedure for the aortic root, can be preferred in appropriate cases [25]. Even though no difference was observed in the cooling, deep hypothermia, and rewarming processes during the application of the modified Bentall technique in the patients considered in our study, this aim can also be achieved in the distal anastomosis form of this technique in appropriate cases [24]. As the reduction of hypothermia levels and circulatory arrest times typically relates to distal anastomosis types, this was not an expected outcome of our study, which focused on the proximal anastomosis technique using the Glue-Felt method. The findings confirmed that there were no significant differences in hypothermia levels and circulatory arrest times between the Glue-Felt and modified Bentall-De Bono techniques. The goal of using the Glue-Felt technique is to avoid fighting on two intense fronts simultaneously, allowing for better allocation of time and effort to the distal anastomosis when needed. Notably, in cases requiring total arch aortic replacement due to distal involvement, the Glue-Felt technique, considered a faster procedure for the aortic root, can be preferred in suitable cases. Although no difference was observed in the cooling, deep hypothermia, and rewarming processes during the application of the modified Bentall technique in our study, the Glue-Felt technique may offer advantages in reducing operative times in complex distal anastomosis cases.

The involvement of the aortic valve, sinotubular junction, fibrous annulus, coronary sinuses, and coronary ostia in acute aortic dissections plays a crucial role in both the patient's expected clinical outcomes and the determination of the type of surgical intervention required. In the ATAAD cases that were included in our study and underwent surgery, cases where there was significant aortic valve insufficiency, or where intimal tears were present in the coronary ostium or coronary sinuses, were considered unsuitable for the Glue-Felt technique and were therefore assigned to the modified Bentall group. In all patients where the Glue-Felt technique was applied, there was no significant aortic valve insufficiency, and no tears were present in the coronary sinuses or ostia. Additionally, during the postoperative echocardiographic evaluations of the Group 1 patients, no cases of aortic insufficiency exceeding mild to moderate levels were observed.

Gluing the aortic root in cases of aortic dissection poses potential risks, including tissue toxicity, aortic root rupture, pseudoaneurysm formation, and possible injury to the aortic valve cusps [28–32]. However, in the Glue-Felt technique we used in our study, these risks were minimized by applying the adhesive material to both sides of the intraoperatively prepared Felt, cut according to the anatomy of the false lumen. During the gluing process, bulldog clamps were used to compress the aortic walls and Felt, ensuring proper adhesion step-by-step, with careful attention to the adherence of the aortic annulus. Additionally, the proximal suture line was placed at the reinforced level of the Glue-Felt interface, which helped minimize these potential risks. In the mid- and long-term follow-ups of our patients, no pseudoaneurysms or ruptures were detected. We believe that the minor variations in the technical details of our method were instrumental in achieving these positive results, contrary to previous reports in the literature [13, 33]. In the statistical analysis, the survival rate of patients using the Glue + Felt technique was significantly higher than that of patients who underwent Bentall-De Bono (*p* < 0.02) (Fig. 2). Considering that there was no difference in the interventions made to the distal aorta, the Glue + Felt technique in the proximal anastomotic site reduces the duration of CPB and ACC. As a result, the development of complications such as acute renal failure, post-operative bleeding, and increased blood transfusion requirement, which may cause the need for hemodialysis due to cardiopulmonary bypass, occurred more frequently in Group-2. These significant advantages in Group-1 contributed to the early period survival (Table 6) (Fig. 2B), which is more evident in the in-hospital and first 30 days' period (Fig. 2A). After propensity score matching, a statistically significant difference in 30-day/in-hospital mortality was also observed between the groups (Fig. 2B). However, among the surviving patients, there was no statistically significant difference in post-discharge mortality rates, both with and without propensity score matching (Figs. 3A, B). The primary aim of comparing the results between the groups is not to demonstrate superiority, as in a randomized trial, but rather to investigate whether the Glue-Felt technique can achieve non-inferior outcomes in carefully selected cases. Considering the scope of surgical options in the ATAAD cases in our study, the choice between the Glue-Felt technique and the modified Bentall-De Bono technique depends largely on several factors, including the degree of aortic root involvement, the extent of aortic valve involvement, the involvement of the coronary ostia, and whether the intimal tear extends into the sinuses of Valsalva. These factors are decisive in determining the surgical approach and, as a result, randomization of patients between the groups was not possible. However, the aim of comparing the two groups was to assess whether the Glue-Felt technique could provide outcomes comparable to the modified Bentall-De Bono aortic root replacement in cases where it is applicable. The findings from our study indicate that the Glue-Felt technique does not demonstrate superiority over the modified Bentall-De Bono procedure, which is considered the gold standard for aortic root replacement. Rather, it shows that the Glue-Felt technique can be a viable alternative, with non-inferior results when applied under appropriate conditions.

In the follow-up of the cases in Group 1, where the Glue-Felt technique was applied, none of the five patients (6.6%) who returned to the hospital required any interventions related to the aortic root or native aortic valve, such as aortic root pseudoaneurysm or additional dissection of the aortic root. It was observed that during the average follow-up period of 46.2 ± 31.6 months, there was no need for re-intervention in these patients. This supports the potential long-term stability of the Glue-Felt technique in suitable cases, contrary to some suggestions in the literature [28–30, 32].

## Conclusion

The primary aim of attempting surgery with the Glue-Felt technique instead of the Bentall-De Bono procedure in ATAAD cases with aortic root involvement is to reduce CPB and ACC times, which are among the leading parameters impacting patient morbidity and mortality. These findings align with the intended hypothetical purpose of the Glue-Felt technique, supporting its use in achieving these goals. While it is true that the Glue-Felt technique is not a universal alternative to aortic root replacement in all cases, it could be considered a potential alternative in situations where aortic root replacement due to dissection may not be necessary. In other words, the modified Bentall procedure, which is the gold standard intervention in cases where the aortic root is dissected, remains a significant method applicable to nearly all such cases. However, in our study, the Glue-Felt technique, though only applicable under certain conditions, is an option that can be considered. Therefore, the Glue + Felt technique used for the proximal anastomotic site in ATAAD surgery can offer significant short-term survival benefit without increasing re-intervention rates.

### Limitations

The retrospective, single-center nature of the study and the limited number of cases are major limitations.

Due to the non-randomizable nature of our study, the distribution of patients between groups reflects the clinical necessity of applying the modified Bentall-De Bono technique, the gold standard for aortic root replacement, in cases where the Glue-Felt technique was not appropriate.

## Supplementary Information

Additional file 1.

## Data Availability

The corresponding author can be contacted to obtain the anonymous dataset used and/or analyzed during the current study upon reasonable request.
